# Phenotypic variation in *Fusarium* crown rot resistance and early-stage salt tolerance among triticale genotypes identifies candidate germplasm for breeding

**DOI:** 10.7717/peerj.21736

**Published:** 2026-09-21

**Authors:** Huihui Wang, Liangxi Zhu, Linyuan Xu, Guanlin Liu, Qing Guo, Yu Sun, Hongjie Li, Yanming Zhang, Lei Cui

**Affiliations:** 1 College of Agriculture, Shanxi Agricultural University, Taiyuan, Shanxi, China; 2 Institute of Biotechnology, Xianghu Laboratory, Hangzhou, Zhejiang, China; 3 Key Laboratory of Molecular Cytogenetics and Genetic Breeding of Heilongjiang Province, College of Life Science and Technology, Harbin Normal University, Harbin, Heilongjiang, China

**Keywords:** Triticale, *Fusarium* crown rot, Salt tolerance, Germplasm screening, Phenotypic variation, Multi-stress adaptation, Cereal breeding

## Abstract

*Fusarium* crown rot (FCR) and soil salinity are major constraints to cereal establishment and productivity worldwide. Triticale (×*Triticosecale* Wittmack), a wheat–rye hybrid, is recognized for its broad adaptation; however, variation in FCR resistance and salinity tolerance among breeding materials remains insufficiently characterized. In this study, 85 triticale genotypes were evaluated for FCR resistance and germination-stage salinity responses, while 49 entries were evaluated for seedling-stage salt tolerance and 48 triticale genotypes were evaluated for agronomic traits. Salinity responses were assessed under 0, 200, 300, and 400 mM NaCl. Significant phenotypic variation was observed for FCR severity, germination performance, seedling salt injury, and agronomic traits. Germination and seedling performance declined with increasing salinity, although the magnitude of reduction differed substantially among genotypes. Several genotypes maintained comparatively high germination under severe salinity and low seedling injury during early vegetative growth, while FCR screening identified lines with reduced disease severity. Correlation and principal component analyses indicated that germination-stage and seedling-stage salinity responses were significantly associated but represented partially distinct aspects of the phenotypic response to salinity. In contrast, FCR resistance showed only weak associations with salinity-tolerance and agronomic traits. Agronomic evaluation under non-stress conditions identified several genotypes that showed favorable phenotypic responses in the FCR and salinity evaluations together with above-average yield-related characteristics. These findings provide a comprehensive phenotypic assessment of FCR resistance, salinity tolerance, and agronomic performance in triticale and identify candidate germplasm for further evaluation in breeding programs targeting multiple production constraints.

## Introduction

Triticale (×*Triticosecale* Wittmack) is a wheat (*Triticum* spp.)–rye (*Secale cereale* L.) hybrid cereal that combinesthe high yield potential and grain quality of wheat with the adaptability, stress tolerance, and vigorous root system of rye (Oettler, 2005). Owing to its broad adaptation to unfavorable environments, triticale has attracted increasing interest as a crop for marginal agricultural lands and low-input production systems (Blum, 2014). However, successful cultivar development requires not only stress tolerance but also favorable agronomic performance, including desirable plant architecture and yield-related traits.

Among the major constraints to cereal production, *Fusarium* crown rot (FCR), primarily caused by *Fusarium pseudograminearum* and related species, is one of the most destructive soil-borne diseases worldwide (Kazan & Gardiner, 2018). FCR reduces tillering, accelerates premature senescence, weakens stem bases, and causes substantial yield losses, particularly in semi-arid cropping systems and conservation-tillage environments (Smiley et al., 2005; Chakraborty et al., 2006; Zhou et al., 2025). Although common wheat is generally susceptible to FCR, triticale has frequently exhibited lower disease severity and has been proposed as a potential source of resistance for cereal improvement programs (Arseniuk & Góral, 2015; Kazan & Gardiner, 2018). Nevertheless, information on the extent of phenotypic variation for FCR resistance within modern triticale breeding populations remains limited.

Soil salinity is another major threat to crop productivity, affecting more than 20% of irrigated agricultural land worldwide (Singh, 2022). Salt stress impairs plant growth through osmotic stress, ion toxicity, and nutrient imbalance, resulting in reduced germination, poor seedling establishment, and decreased biomass accumulation (Ashraf & Munns, 2022; Liang et al., 2024). Germination and early seedling growth are particularly sensitive to salinity because these developmental stages directly influence stand establishment and subsequent crop performance. Consequently, screening at these stages is widely used to characterize phenotypic variation in salinity responses and to support early-generation selection in cereal breeding programs (Munns & Tester, 2008; Ashraf & Foolad, 2013).

Although salinity tolerance has been extensively studied in wheat, rye, barley (*Hordeum vulgare* L.), and oat (*Avena sativa* L.) (Hussain et al., 2021; Gharaghanipor et al., 2022; Singh & Roychoudhury, 2022; Zhang et al., 2022), relatively few studies have evaluated salinity responses in diverse triticale germplasm collections. Existing investigations have often focused on physiological traits, biomass responses, or a limited number of genotypes (Salehi & Arzani, 2013; Mohammadi Alagoz et al., 2023; Rasouli et al., 2024; Abd El Baki et al., 2025). Studies of triticale have generally examined either disease resistance or abiotic stress tolerance separately (Arseniuk & Góral, 2015; Ramadan et al., 2023). As a result, there is limited information on the simultaneous characterization of FCR resistance, salinity tolerance, and agronomic performance within the same breeding population, making it difficult to identify germplasm with multiple favorable traits for breeding applications.

Abiotic and biotic stresses frequently occur in the same production environments, particularly in semi-arid regions where soil salinity and FCR can jointly limit crop establishment and productivity. Therefore, breeding programs benefit from the identification of germplasm exhibiting favorable phenotypic responses to both stresses while maintaining acceptable agronomic characteristics. However, comprehensive evaluations integrating these traits in triticale remain limited.

The present study addressed this gap by evaluating 85 triticale genotypes for FCR resistance and germination-stage salinity responses, together with seedling-stage salinity responses and agronomic traits evaluated in subsets of the germplasm. The objective was to identify candidate germplasm exhibiting favorable phenotypic responses in separate disease-resistance and salinity-tolerance screening assays together with desirable agronomic characteristics. The resulting phenotypic dataset provides a valuable resource for identifying candidate germplasm and supporting future triticale improvement efforts.

## Materials & Methods

### Plant materials

A total of 85 triticale genotypes was evaluated in this study. These consisted of 31 advanced breeding lines designated J23, four breeding lines designated Z23, and 28 breeding lines designated G23 developed within our breeding program, together with 27 additional triticale germplasm accessions (advanced breeding lines and released varieties) obtained from research institutes and breeding programs in China. Detailed information for all entries, including genotype designation, origin, germplasm status, and source, is provided in Table S1. The salt-tolerant wheat cultivar CDH was included as a reference control in the salinity experiments only and was not included in the FCR evaluation. Four rye (*S. cereale*) varieties (W23-3, WA-3, WA8, and WA17) were included as reference controls in both the salinity and FCR experiments. Unless otherwise specified, the statistical analyses for each experiment included all entries evaluated in the corresponding assay, including the appropriate reference controls.

### Seedling reactions to FCR disease

The FCR assays were conducted using *F. pseudograminearum* strain WZ-8A, a prevalent and aggressive isolate from the Yellow and Huai River wheat regions of China. For inoculum preparation, 500 g of millet grains were boiled for 3 min, drained, rinsed with cold water, and surface-dried on sterile gauze. After sterilizing by autoclaving at 121 °C for 30 min and cooling, grains were inoculated with 7–10 mycelial plugs (∼5 mm diameter) of strain WZ-8A per bag. Inoculated grains were incubated in darkness at 25 °C for 7 d with daily shaking, air-dried following full colonization, and stored at room temperature until use.

### Seeding inoculation and disease assessment

Seeds were surface-disinfested in 0.5% hydrogen peroxide for 30 min, rinsed thoroughly with sterile water, and sown in sterilized soil:vermiculite (2:1, w/w). Each genotype was replicated three times with 15 plants per replicate. At one-leaf stage, a single infested millet grain was placed in contact with the crown of each seedling; control plants received a sterile grain. Plants were grown at 25/15 °C (day/night) under a 14-h photoperiod and evaluated 4 weeks post-inoculation. Crown rot severity was scored on a 0–9 scale (0 = no symptoms; 9 = severe to complete necrotic) (Li et al., 2008; Yang et al., 2019). A disease index (DI) was calculated for each genotype per replicate using the following formula (Li et al., 2008; Zheng et al., 2014):

\begin{eqnarray*}DI= \frac{\sum ({n}_{i}\times {s}_{i})}{N\times S} \times 100 \end{eqnarray*}

where *n*_*i*_ is the number of plants assigned to severity score *i*, *s*_*i*_ is the corresponding disease severity score, *N* is the total number of plants assessed, and *S* is the maximum disease severity score. The DI value is expressed as a percentage, with higher values indicating more severe FCR symptoms. Based on the DI, genotypes were classified into four reaction categories for descriptive purposes: highly resistant (HR), moderately resistant (MR), moderately susceptible (MS), and highly susceptible (HS). Classification thresholds were defined as follows: HR (DI ≤ 10), MR (11 < DI ≤ 20), MS (21 < DI ≤ 30), and HS (DI > 31).

### Germination and seedling growth under salt treatment

Seeds were surface-sterilized in 1% NaOCl for 30 min and rinsed thoroughly with distilled water. For germination assays, three independent Petri dish replicates were prepared for each genotype × treatment combination, with 30 seeds per replicate dish. Seeds were placed on filter paper in 9-cm Petri dishes and subjected to three salinity levels: 0 (control), 200 (moderate), and 300 mM NaCl (severe). The selected NaCl concentrations were used to generate a broad range of stress intensities and to maximize discrimination among genotypes. While the higher concentrations exceed salinity levels commonly encountered in cereal production systems, they are frequently employed in controlled-environment screening studies to identify genetic variation in tolerance to severe osmotic and ionic stress. Dishes were incubated at 20 ± 2 °C under a 16-h light/8-h dark photoperiod for 7 d. Filter papers were replaced every 2 d to avoid salt accumulation. Germination was recorded after 7 d, defined as radicle emergence ≥1 mm.

Seeds were surface-sterilized in 75% (v/v) ethanol for 3 min and rinsed with distilled water for 50 s. After germination on moistened filter paper for 7 d in a growth chamber (16 h light, 20 °C/8 h dark, 16 °C), uniform seedlings at the two-leaf stage were transferred to 96-well hydroponic trays containing Hoagland nutrient solution. For each genotype, five seedlings per genotype were exposed to nutrient solutions containing 0, 300, or 400 mM NaCl in the nutrient solution, with three biological replicates per treatment. Seedling phenotypes were recorded after 7 d of salt stress.

For the germination-stage salinity experiments, all 90 entries (85 triticale genotypes, one wheat reference cultivar, and four rye reference varieties) were included in the analysis. For the seedling-stage salinity experiment, salt treatment was imposed only after seedlings had reached the two-leaf stage. Only entries with at least five healthy and uniform seedlings reaching the two-leaf stage were included in the seedling-stage evaluation, whereas entries that did not meet this criterion before salt treatment were excluded. Consequently, 49 entries were included in the seedling-stage salinity analysis. Because salt stress was applied only after seedlings had reached the two-leaf stage, these exclusions reflected insufficient seedling establishment under the experimental conditions rather than responses to salt treatment.

### Germination and seedling parameters

The following parameters were calculated:

Germination percentage (GP%) was calculated as previously described (Manmathan & Lapitan, 2013):

\begin{eqnarray*}GP \left( \% \right) = \frac{{N}_{g}}{{N}_{t}} \times 100 \end{eqnarray*}

where *N*_*g*_ is the number of germinated seeds, and *N*_*t*_ is the total number of seeds tested. GP was calculated separately for each biological replicate.

To account for genotypic differences in baseline germination, germination under salt stress was normalized to the control and expressed as relative germination rate (RGR):

\begin{eqnarray*}RGR= \frac{G{P}_{salt}}{G{P}_{control}} \end{eqnarray*}

where *GP*_*salt*_ and *GP*_*control*_ are germination percentages under salt treatment (200 or 300 mM NaCl) and control (0 mM NaCl), respectively. RGR was subsequently calculated for each replicate using the corresponding control and salt-treatment GP values, and mean values ± standard deviations were calculated from the three biological replicates.

Relative salt injury (RSI) (Ramadan et al., 2023) was used to assess salt-induced reduction in germination. RSI was calculated based on germination percentage using the following formula:

\begin{eqnarray*}RSI= \frac{G{P}_{control}-G{P}_{salt}}{G{P}_{control}} \end{eqnarray*}

where *GP*_*salt*_ and *GP*_*control*_ are germination percentages under salt stress and control conditions, respectively. RSI was calculated separately for each biological replicate using the corresponding GP values under control and salt-stress conditions. Mean values ± standard deviations were then calculated from the three biological replicates. Lower RSI values indicate higher salt tolerance. Because RSI is mathematically complementary to RGR (RSI = 1 − RGR), statistical analyses were performed on RGR only. RSI is presented as an alternative expression of salt-induced germination reduction to facilitate comparison with previous salinity-screening studies.

Seedling-stage salt tolerance was evaluated using a visual salt-injury scoring method based on a 0–9 injury scale as described by Gregorio, Senadhira & Mendoza (1997), where zero indicates no visible injury and nine indicates severe injury or plant death. The seedling-stage salt injury index (SII) was calculated using the following formula:

\begin{eqnarray*}SII= \frac{\sum ({n}_{i}\times {g}_{i})}{N\times G} \end{eqnarray*}

where *n*_*i*_ is the number of seedlings assigned to injury grade *i*, *g*_*i*_ is the corresponding injury grade value, *N* is the total number of seedlings evaluated, and *G* is the maximum injury grade. Lower SII values indicate higher salt tolerance, whereas higher values indicate increased sensitivity to salt stress.

### Evaluation of agronomic traits

Agronomic evaluation was conducted in an independent field experiment comprising 48 triticale genotypes for which sufficient seed was available for field planting. These entries were not selected on the basis of their responses in the FCR or salinity experiments. Plant height (PH) was recorded at maturity (Zadoks growth stage 90, GS90), measured from the ground level to the spike tip excluding awns. Spike length (SL) was measured from the base of the rachis to the tip of the spike. The number of grains per spike (NGS) was counted. Each trait was measured from five randomly selected plants per genotype. Spikes were manually threshed using a bench micro-thresher to determine the thousand-kernel weight (TKW).

### Statistical analysis

All experiments were conducted using three biological replicates. Data are presented as mean ± standard deviation (SD). For the FCR assay, differences in DI among genotypes were evaluated using one-way analysis of variance (ANOVA), with genotype treated as a fixed effect. For germination assays, GP was analyzed using a generalized linear model (GLM) with a binomial error distribution and logit link function. The numbers of germinated and non-germinated seeds per Petri dish were used as the response variable, and genotype, salt treatment, and their interaction were included as fixed effects. Wald chi-square statistics were used to assess the significance of model terms. Relative germination rate (RGR) and RSI were calculated for each biological replicate using the corresponding control and salt-treatment germination percentages. Differences in RGR among genotypes and salt treatments were evaluated using two-way analysis of variance, with genotype, salt treatment, and their interaction treated as fixed effects. Seedling-stage salt injury index (SII) was similarly analyzed using two-way ANOVA, with genotype, salt treatment, and genotype × treatment interaction included as fixed effects. Because RSI is mathematically complementary to RGR, statistical analysis was performed on RGR only. Agronomic traits, including PH, SL, NGS, and TKW, were analyzed using one-way ANOVA with genotype as a fixed effect. When significant genotype effects were detected, means were separated using Tukey’s honestly significant difference (HSD) test at *P* < 0.05. Pearson correlation analysis was performed to examine relationships among Fusarium crown rot disease index, salinity-tolerance traits, and agronomic characteristics. Correlation coefficients were calculated using pairwise complete observations to account for missing values among trait groups. Correlation matrices were visualized as heatmaps, and significance levels were determined using Pearson’s correlation tests. Principal component analysis (PCA) was conducted to summarize multivariate relationships among disease-resistance, salinity-tolerance, and agronomic traits. To avoid over-representation of highly correlated variables, trait selection was guided by Pearson correlation analysis. Seven representative traits were retained for PCA: *Fusarium* crown rot disease index (FCR DI), relative germination rate at 300 mM NaCl (RGR300), seedling-stage salt injury index at 400 mM NaCl (SII400), PH, SL, NGS, and TKW. Traits exhibiting strong redundancy, including GR200, GR300, RGR200, and SII300, were excluded because they were highly correlated with retained variables. Trait values were standardized to zero mean and unit variance before analysis. Principal components were calculated from the correlation matrix, and genotype distributions and trait loadings were visualized using a PCA biplot. All statistical analyses were performed in R (version 4.4.0; R Core Team, 2024). Statistical significance was declared at *P* < 0.05.

## Results

### Variation in FCR responses among the evaluated germplasm

**Figure 1:**
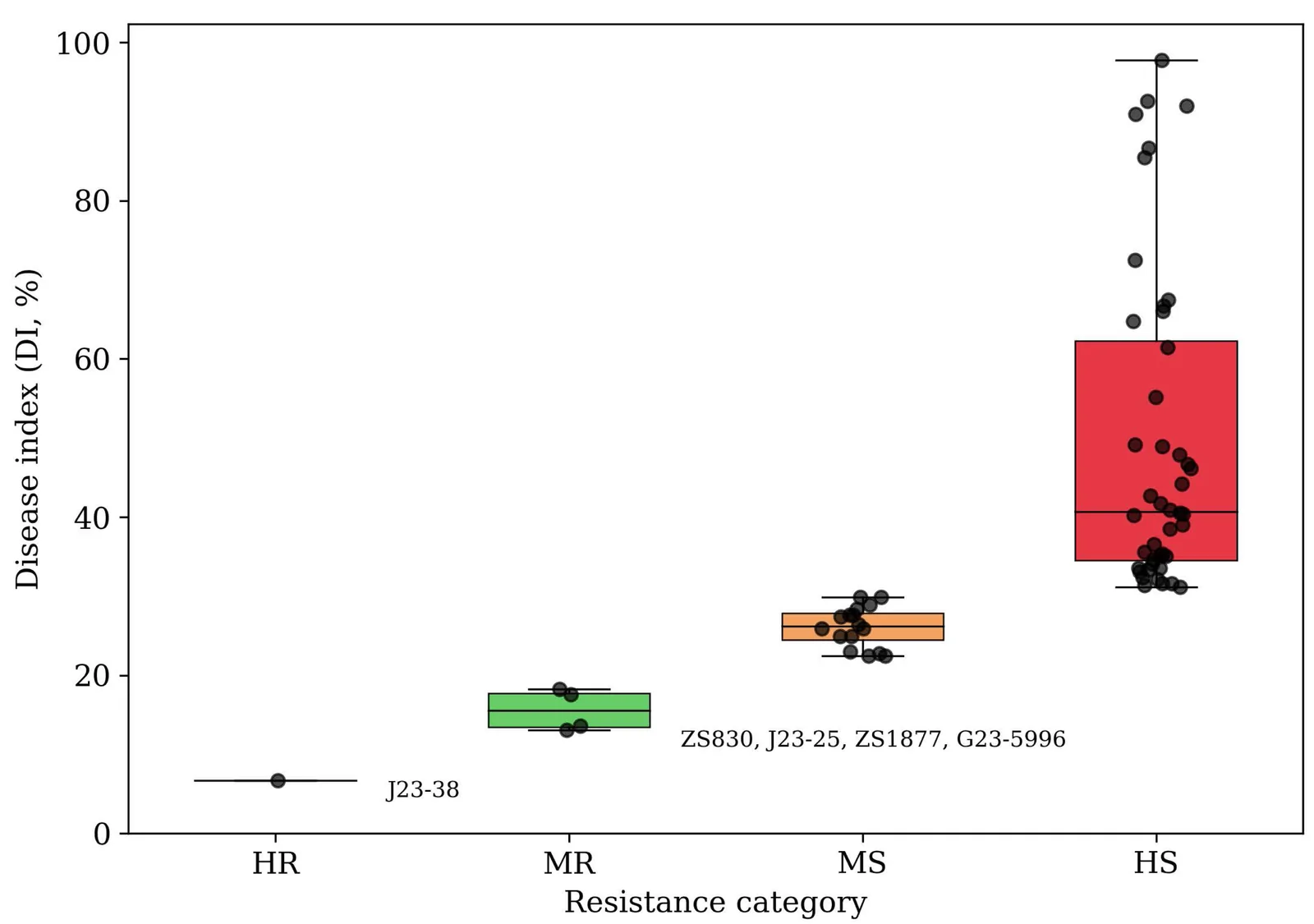
Variation of *Fusarium* crown rot resistance among triticale genotypes. Boxplots show the distribution of mean DI values for genotypes classified as highly resistant (HR), moderately resistant (MR), moderately susceptible (MS), and highly susceptible (HS). The box indicates the interquartile range, the interior line represents the median, and whisker denotes the value range. Dots represent individual genotype means. HR and MR genotypes are labeled.

One-way analysis of variance (ANOVA) revealed a highly significant genotype effect on DI (*P* < 0.001; Table S2), indicating substantial variation in FCR resistance among the evaluated genotypes. The DI values ranged from 6.67 to 97.78, demonstrating a broad spectrum of responses to FCR infection (Fig. 1). Based on DI values, the genotypes were classified into four resistance categories: HR, MR, MS, and HS. Most genotypes were classified as HS, whereas only a limited number showed HR or MR reactions. Line J23-38 exhibited the highest resistance (DI = 6.67). Genotypes ZS830, J23-25, ZS1877, and G23-5996 displayed MR, with DI values ranging from 13.09 to 18.27.

Several genotypes were classified as MS, displaying intermediate DI values under experimental conditions. Most genotypes, however, were HS. Among these, genotypes such as J23-28, G23-5521, J23-15, and G23-5811 showed extensive necrosis, with mean DI scores exceeding 90%.

### Germination-stage salinity responses among the evaluated entries

Absolute GP under control and salt stress conditions is presented in Fig. S1. Under control conditions (0 mM NaCl), GP ranged from 62% to 99%, indicating substantial variation in baseline seed germinability among triticale genotypes. Germination percentage declined progressively with increasing salinity, although the magnitude of reduction differed among genotypes. Generalized linear model analysis revealed significant effects of genotype (*χ*^2^ = 645.56, *P* < 0.001), salt treatment (*χ*^2^ = 2,979.47, *P* < 0.001), and genotype × salt treatment interaction (*χ*^2^ = 391.50, *P* < 0.001) on germination percentage (Table S3). These results indicate substantial phenotypic variation in germination responses and demonstrate that genotypes differed significantly in their responses to increasing salinity stress. Genotypic rankings based on GP under salt stress often differed from those observed under control conditions. For example, J23-30 exhibited the lowest GP under control conditions (62%) but maintained 37% germination at 300 mM NaCl, exceeding that of G23-5911 (35%; control GP, 90%) and J23-32 (34%; control GP, 97%). This discrepancy between baseline germination capacity and germination under salinity stress highlights the importance of normalized indices for comparing phenotypic responses to salinity among genotypes with differing germination potential.

**Figure 2:**
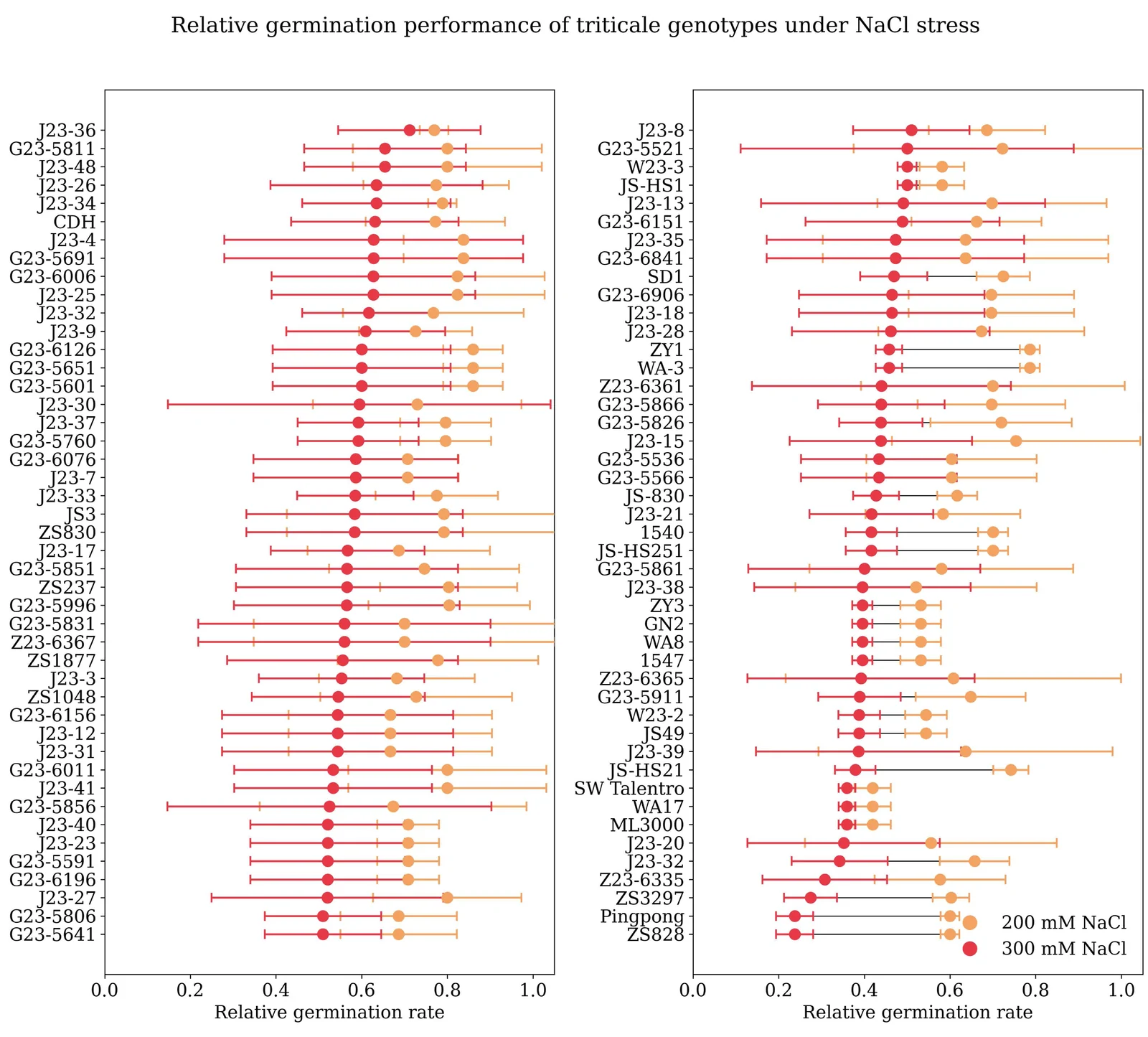
Salt stress effects on triticale germination. Data points show mean ± s.d. (*n* = 3). Paired lines link values for each genotype across salt levels. Genotypes are ranked by relative germination rate at 300 mM NaCl.

Relative germination rate, which normalizes germination under salt stress to the corresponding control, revealed substantial variation in salt tolerance at both 200 mM and 300 mM NaCl (Fig. 2). Two-way ANOVA showed significant effects of genotype (*F* = 38.85, *P* < 0.001), salt treatment (*F* = 22.31, *P* < 0.001), and their interaction (*F* = 1.75, *P* < 0.001) (Table S4). These results indicate that genotypes differed significantly in their ability to maintain germination under salinity stress and that the magnitude of the response varied with salt concentration. At 200 mM NaCl, genotypes G23-6126, G23-5651, G23-5601, J23-4, and G23-5691 exhibited the highest RGR values, indicating minimal inhibition of germination relative to the control. Under 300 mM NaCl, differences among genotypes became more pronounced. J23-36 showed the highest RGR, followed by G23-5811, J23-48, J23-26, J23-34, J23-4, and G23-5691, indicating superior maintenance of germination under severe salinity stress.

Because relative salt injury is mathematically complementary to RGR (RSI = 1 − RGR), both indices produced identical genotype rankings. Therefore, RSI is presented as an alternative representation of germination reduction under salinity stress rather than as an independent measure of salt tolerance (Fig. S2). Consistent with the RGR results, genotypes exhibiting high RGR displayed correspondingly low RSI values. At 200 mM NaCl, J23-36, J23-4, G23-5691, and the salt-tolerant wheat control CDH showed among the lowest RSI values. At 300 mM NaCl, J23-48, G23-5811, and J23-34 maintained comparatively low RSI values, indicating limited additional inhibition of germination under severe salinity stress.

Overall, two general patterns of germination responses were observed. First, several genotypes, including J23-36, J23-48, and G23-5691, consistently exhibited high RGR and comparatively low RSI under salinity stress, indicating favorable germination performance across multiple salinity-response metrics. Second, genotypes such as G23-5811, J23-48, and J23-36, exhibited relatively small changes in RGR and RSI between 200 and 300 mM NaCl, indicating comparatively stable responses across increasing salinity levels.

### Seedling-stage salinity responses among the evaluated germplasm

**Figure 3:**
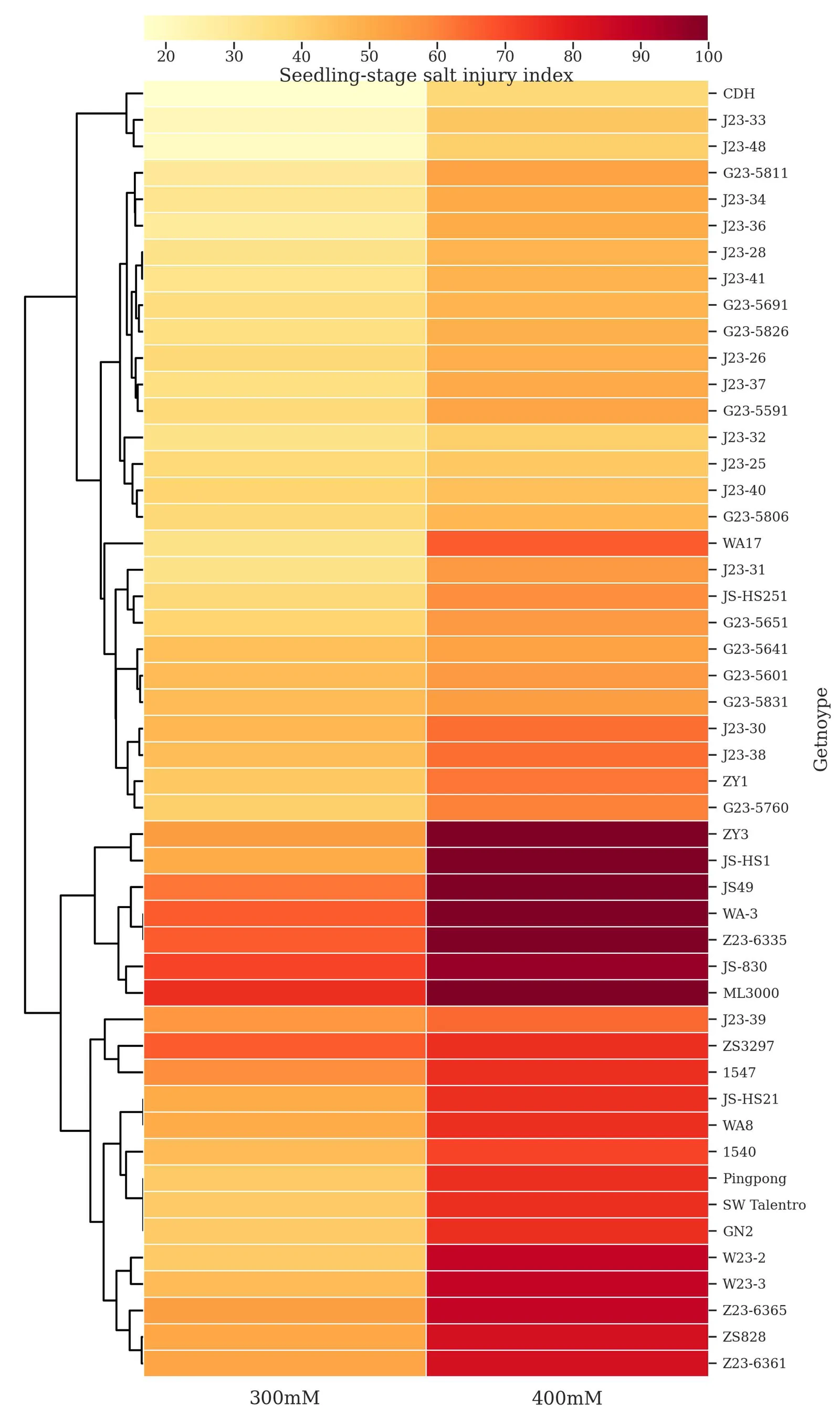
Seedling-stage salt injury under 300 mM and 400 mM NaCl stress. Clustered heatmap shows injury indices of triticale genotypes at 300 and 400 mM NaCl. Genotypes are hierarchically clustered by response. Color gradient indicates injury level (yellow, low; red, high). Axes label genotypes (y) and treatments (x).

Seedling-stage salt injury index was evaluated for the 49 entries that produced sufficient healthy and uniform seedlings to reach the two-leaf stage for salt treatment. Substantial variation in SII during early vegetative growth was observed among these entries (Fig. 3). Two-way ANOVA showed significant effects of genotype (*F* = 156.74, *P* < 0.001), salt treatment (*F* = 3,868.59, *P* < 0.001), and genotype × salt treatment interaction (*F* = 19.05, *P* < 0.001) on SII (Table S5). These results indicate that genotypes differed significantly in the extent of salt-induced injury and in their responses to increasing salinity.

Salt injury increased with stress intensity, ranging from 16.67–75.00 at 300 mM NaCl and from 37.50–100.00 at 400 mM NaCl. The heatmap visualization highlighted substantial differences among genotypes across both salinity levels (Fig. 3). Several genotypes maintained comparatively low injury under both treatments, particularly under severe stress. At 400 mM NaCl, the lowest SII values were observed in the salt-tolerant wheat control CDH (37.50), followed by J23-48 (40.15), J23-32 (40.17), J23-25 (42.13), J23-33 (42.63), J23-40 (44.63), G23-5806 (46.50), G23-5691 (47.43), J23-28 (47.55), and J23-41 (47.95). In contrast, several genotypes exhibited severe injury under 400 mM NaCl. ZY3, JS-HS1, ML3000, JS49, WA-3, and Z23-6335 reached the maximum SII value of 100.00, while JS-830, W23-2, W23-3, and Z23-6365 also exhibited high injury levels.

Rankings based on seedling-stage SII differed from those obtained from germination-stage assessments. Several genotypes that showed moderate germination-stage performance maintained relatively low injury during early vegetative growth under severe salinity. For example, J23-28 and G23-5826 exhibited comparatively weaker performance during germination but showed low SII values at 400 mM NaCl. Pearson correlation analysis further demonstrated that germination-stage and seedling-stage salinity responses were significantly associated but not strongly correlated (Fig. S3). Relative germination rate at 300 mM NaCl was negatively correlated with SII at 400 mM NaCl (*r* = −0.29, *P* < 0.05), indicating that genotypes maintaining higher germination under severe salinity generally sustained lower seedling injury. However, the moderate strength of this relationship and the observed changes in genotype rankings between developmental stages indicate that salinity responses during germination and early vegetative growth represent partially distinct aspects of the phenotypic response to salinity.

### Agronomic variation among triticale genotypes

**Figure 4:**
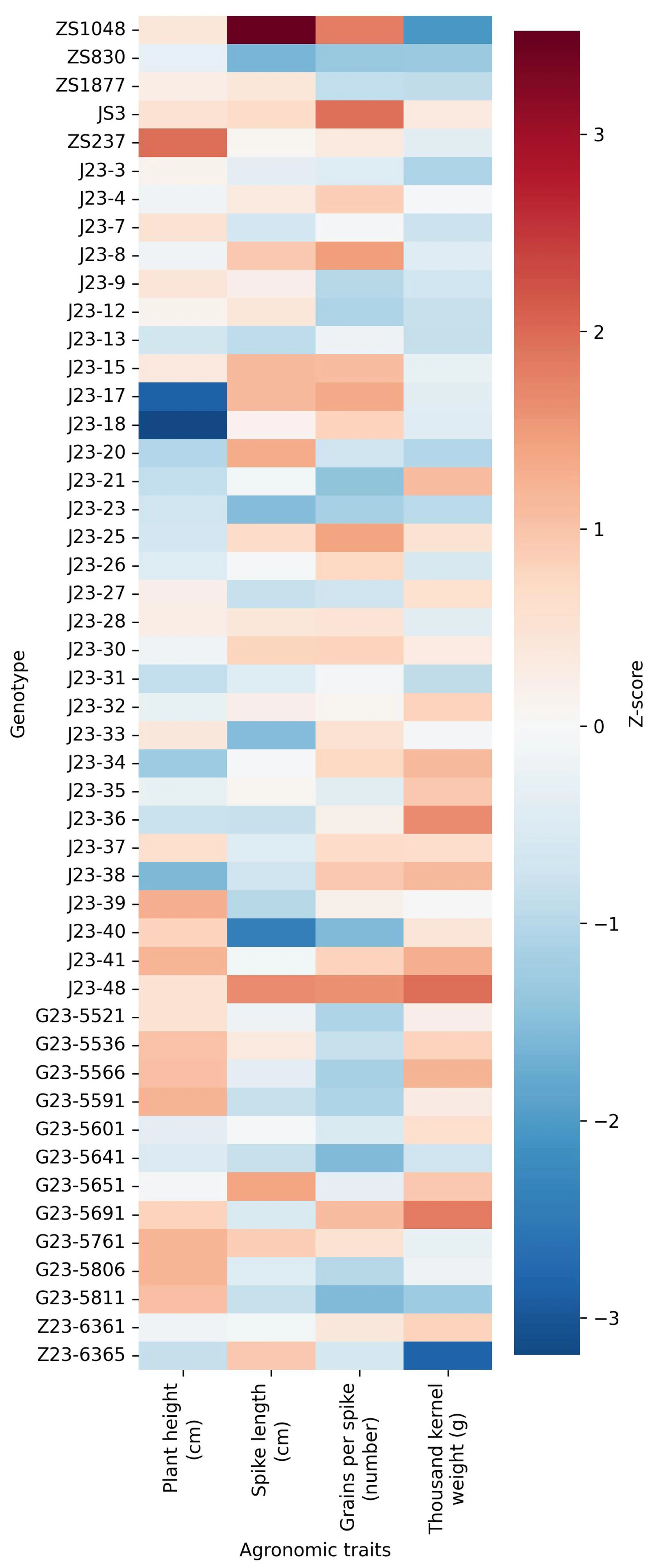
Agronomic trait heatmap of triticale genotypes. Z-scores indicate each genotype’s trait performance relative to the population mean. Red and blue denote above- and below-average values, respectively.

To evaluate the agronomic performance, PH, SL, NGS, and TKW were assessed under non-stress conditions. Analysis of variance revealed significant genotypic effects for all agronomic traits examined (Table S6), indicating substantial phenotypic variation among the evaluated triticale genotypes. Heatmap visualization further illustrated variation in agronomic trait profiles across the germplasm panel (Fig. 4). Plant height varied from 132.0 cm in J23-18 to 177.6 cm in ZS237 (*P* < 0.05). Other tall genotypes included J23-39 (171.6 cm), G23-5591 (171.0 cm), G23-41 (170.8 cm), G23-5761 (170.8 cm), and G23-5806 (170.8 cm). Spike length also differed significantly among genotypes and was not strictly associated with plant height. ZS1048 exhibited the longest spikes (15.8 cm; *P* < 0.05), while J23-48 (13.7 cm), G23-5651 (13.4 cm), and J23-15 (13.1 cm) also exceeded the population mean. In contrast, J23-23 (10.1 cm), ZS830 (10.0 cm), and J23-40 (9.1 cm) exhibited shorter spikes. Substantial variation was also observed for yield-related traits. Grain number per spike ranged from 22.0 to 32.4, with JS3 (32.4 grains), J23-48 (31.4 grains), J23-8 (31.0 grains), and G23-5691 (29.8 grains) ranking among the highest-performing genotypes. Thousand-kernel weight ranged from 28.56 to 46.90 g. J23-48 (46.90 g), G23-5691 (46.36 g), J23-36 (45.66 g), and J23-41 (44.26 g) exhibited the highest TKW values, whereas Z23-6365 (28.56 g) and ZS1048 (31.60 g) had comparatively lower kernel weights.

Pearson correlation analysis revealed significant relationships among several agronomic traits (Fig. S3). The strongest association was observed between SL and NGS (*r* = 0.50, *P* < 0.001), indicating that genotypes with longer spikes generally produced more grains per spike. Other trait associations were comparatively weak to moderate.

When agronomic performance was considered together with the separate evaluations of FCR resistance and salinity tolerance, several genotypes, including J23-48 and G23-5691, exhibited favorable phenotypic performance across the individual assessments while maintaining above-average values for key yield-related traits under non-stress conditions. These genotypes therefore represent candidate germplasm for further evaluation in breeding programs.

### Associations among FCR resistance, salinity tolerance, and agronomic traits

Pearson correlation analysis revealed several significant relationships among germination-stage salinity traits, seedling-stage SII, and agronomic characteristics (Fig. S3). Germination percentages under 200 mM (GR200) and 300 mM NaCl (GR300) were strongly positively correlated (*r* = 0.82, *P* < 0.001), indicating that genotypes exhibiting high germination under moderate salinity generally maintained superior germination under more severe salinity. Similarly, seedling-stage salt injury indices at 300 mM (SII300) and 400 mM NaCl (SII400) were strongly positively correlated (*r* = 0.83, *P* < 0.001), indicating consistent genotypic responses across the two seedling-sate salinity treatments.

Germination-stage and seedling-stage salinity responses were significantly associated but not identical. Germination percentages under salinity stress were negatively correlated with both SII300 and SII400 (*r* = −0.62 to −0.72, *P* < 0.001), indicating that genotypes maintaining higher germination under salt stress generally sustained less injury during subsequent seedling development. Relative germination rates showed similar relationships, being positively associated with germination under salinity and negatively associated with seedling-stage injury indices. However, the correlations were moderate rather than strong, suggesting that salinity responses during germination and early vegetative growth represent related but partially distinct aspects of the phenotypic response to salinity.

In contrast, Fusarium crown rot disease index exhibited weak and generally non-significant correlations with salinity-tolerance and agronomic traits, indicating that variation in FCR response was largely independent of variation in salinity responses and agronomic performance within the evaluated germplasm panel.

Among agronomic traits, spike length was positively correlated with grain number per spike (*r* = 0.50, *P* < 0.001), indicating that genotypes with longer spikes generally produced more grains per spike. In addition, SII400 was negatively correlated with thousand-kernel weight (*r* = −0.43, *P* < 0.05), indicating that genotypes exhibiting lower injury under severe salinity tended to possess higher kernel weight under non-stress conditions. Most other agronomic traits showed weak associations with disease-resistance and salinity-tolerance traits.

### Integrated variation in FCR resistance, salinity tolerance, and agronomic traits

**Figure 5:**
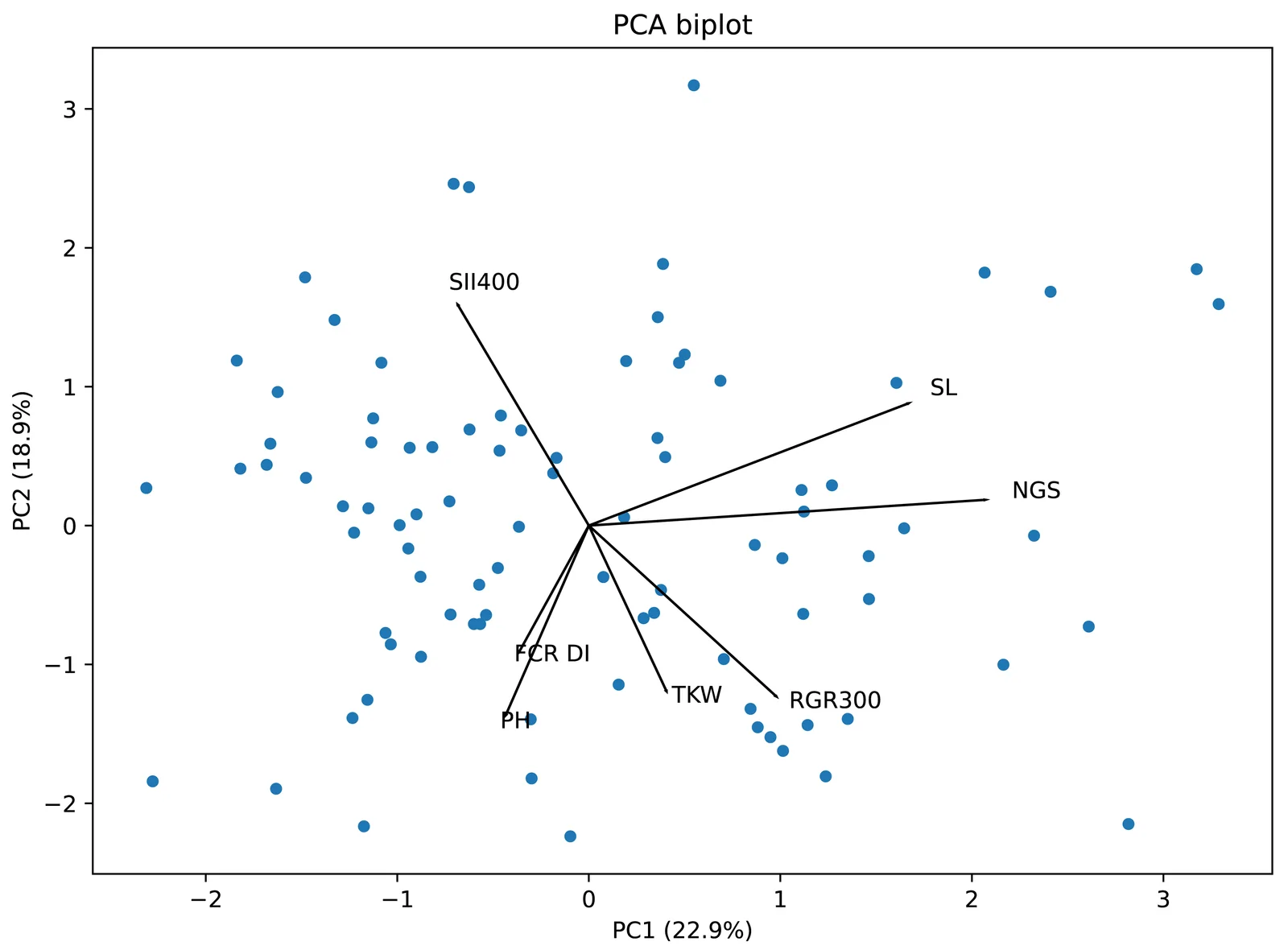
Principal component analysis of *Fusarium* crown rot resistance, salinity-tolerance, and agronomic traits in 85 triticale genotypes. The analysis was performed using seven representative traits selected from Pearson correlation analysis: *Fusarium* crown rot disease index (FCR DI), relative germination rate at 300 mM NaCl (RGR300), seedling-stage salt injury index at 400 mM NaCl (SII400), plant height (PH), spike length (SL), number of grains per spike (NGS), and thousand-kernel weight (TKW).

To summarize multivariate relationships among FCR resistance, salinity tolerance, and agronomic traits, PCA was performed using seven representative variables selected on the basis of the Pearson correlation analysis (Fig. 5). Trait selection was intended to minimize redundancy among highly correlated variables while retaining key indicators of FCR resistance (FCR DI), germination-stage salinity tolerance (RGR300), seedling-stage salinity tolerance (SII400), and agronomic performance (PH, SL, NGS, and TKW). Variables exhibiting strong pairwise correlations, including GR200, GR300, RGR200, and SII300, were excluded because they provided largely overlapping information with the retained traits.

The first two principal components explained 41.8% of the total phenotypic variation, with PC1 and PC2 accounting for 22.9% and 18.9%, respectively. PC1 was primarily associated with agronomic traits, particularly SL and NGS, which exhibited the largest positive loadings. In contrast, PC2 was influenced mainly by salinity-response traits, with seedling-stage salt injury (SII400) loading positively and relative germination rate under severe salinity (RGR300) loading negatively. Plant height and TKW also contributed to variation along PC2.

The PCA biplot indicated that agronomic traits and salinity-response traits represented largely distinct dimensions of phenotypic variation. Consistent with the Pearson correlation analysis, FCR disease index exhibited relatively small loadings on both principal components and showed limited association with salinity-tolerance and agronomic traits. These results suggest that variation in FCR response was largely independent of variation in salinity responses and agronomic performance within the evaluated germplasm panel.

## Discussion

Substantial phenotypic variation in salinity responses was observed among the evaluated triticale germplasm, demonstrating considerable diversity in responses to salt stress during early development. Salinity imposes osmotic stress, ion toxicity, and oxidative damage, all of which can impair germination and seedling establishment (Melino & Tester, 2023). Consistent with previous studies in wheat and barley (Hussain et al., 2021; Gharaghanipor et al., 2022), the present results confirm the existence of broad variation in salinity responses within triticale germplasm. Several breeding lines, particularly within the J23 and G23 groups, maintained comparatively favorable performance under salinity stress, supporting their further evaluation as candidate germplasm for breeding programs targeting salt-affected environments.

Seed germination is particularly sensitive to salinity because elevated salt concentrations restrict water uptake, disrupt cellular metabolism, and induce oxidative damage in embryonic tissues (El Moukhtari et al., 2023). Accordingly, germination percentage declined progressively with increasing NaCl concentration, with substantial inhibition observed at 300 mM NaCl. Similar responses have been reported in wheat and barley under severe salinity stress (Awad et al., 2021). Despite this inhibition, several triticale genotypes maintained comparatively high germination under 300 mM NaCl, demonstrating substantial phenotypic variation in germination-stage salinity responses. The capacity to maintain germination under saline conditions is important for crop establishment because successful emergence determines subsequent stand development and uniformity. Because the salinity treatments were imposed under controlled conditions and included stress levels exceeding those typically encountered in agricultural production systems, the observed responses should be interpreted as indicators of relative salinity tolerance rather than direct predictors of field performance. Nevertheless, these conditions provided strong discrimination among genotypes and enabled the identification of candidate germplasm for subsequent evaluation under field-relevant salinity environments.

Seedling-stage salt injury further differentiated genotypes according to their responses during early vegetative growth. Several genotypes that exhibited only moderate germination-stage performance, including J23-28 and G23-5826, maintained comparatively low injury under severe salinity, indicating favorable post-germination responses. Pearson correlation analysis demonstrated that germination-stage and seedling-stage salinity traits were significantly associated, whereas principal component analysis indicated that they contributed to different dimensions of phenotypic variation. Together, these results indicate that salinity responses during germination and early vegetative growth are related but represent distinct aspects of the phenotypic response to salinity. Similar conclusions have been reported in wheat and barley, where previous studies suggest that germination- and seedling-stage responses involve partially distinct physiological mechanisms (Roy, Negrão & Tester, 2014; Oyiga et al., 2016; Al-Ashkar et al., 2020).

The physiological basis of these stage-dependent responses remains beyond the scope of the present study but is likely related to mechanisms operating after germination, including Na^+^ exclusion from young leaves, vacuolar ion sequestration, maintenance of K^+^/Na^+^ homeostasis, and preservation of photosynthetic activity under saline conditions (Munns et al., 2012; Gupta & Huang, 2014). Enhanced antioxidant capacity and membrane stability may further contribute to reduced tissue injury during prolonged salt exposure (Hasanuzzaman et al., 2021). Consequently, screening programs based solely on germination performance may overlook genotypes possessing favorable phenotypic responses during subsequent stages of development.

In addition to salinity tolerance, resistance to FCR remains an important breeding target in cereal production systems exposed to multiple stresses (Kazan & Gardiner, 2018). The FCR bioassays effectively differentiated genotypes according to disease severity, and several triticale lines exhibited comparatively low disease indices. Notably, genotypes such as J23-25 and G23-5996 combined low FCR severity with favorable salinity responses. Correlation and PCA analyses further demonstrated that FCR disease index exhibited only weak associations with salinity-response and agronomic traits. This pattern suggests that FCR resistance and salinity responses largely represent independent dimensions of phenotypic variation within the evaluated germplasm, indicating that these traits may be improved concurrently through breeding. Such integration is important because both FCR and salinity can substantially reduce grain yield and yield components under production conditions (Smiley et al., 2005; Munns & Tester, 2008; Munns & Gilliham, 2015; Kazan & Gardiner, 2018). However, because FCR resistance and salinity tolerance were evaluated in separate controlled-environment experiments, confirmation under combined-stress conditions will be required.

The breeding value of stress-responsive germplasm ultimately depends on its compatibility with favorable agronomic characteristics. Agronomic evaluation under non-stress conditions identified several genotypes, including J23-36, J23-48, J23-25, and G23-5691, that combined favorable phenotypic responses in the salinity-tolerance and FCR-resistance assays with above-average yield-related traits. Correlation analysis revealed a positive association between SL and NGS, whereas severe seedling salt injury was negatively associated with TKW. These relationships suggest that certain agronomic traits were associated with salinity responses, although the overall correlations were generally weak. Therefore, disease resistance, salinity responses, and agronomic performance appeared to represent largely independent dimensions of phenotypic variation within the germplasm panel.

Nevertheless, agronomic traits were evaluated only under non-stress conditions, and neither salinity tolerance nor FCR resistance was assessed under field environments. Consequently, the identified genotypes should be regarded as promising candidate breeding materials rather than confirmed sources of field-level resilience or yield stability under salinity and disease pressure. Future studies should evaluate agronomic performance under salinity stress, FCR infection, and combined-stress scenarios to determine yield stability, trait plasticity, and stress interactions. Multi-environment field trials will be essential for validating the breeding value and adaptation of these genotypes under target production conditions.

## Conclusions

This study revealed substantial phenotypic variation in FCR resistance and germination-stage salinity responses among the 85 triticale genotypes, while seedling-stage salt tolerance and agronomic performance were evaluated in subsets of 49 and 48 triticale genotypes, respectively. Several genotypes exhibited favorable phenotypic responses in the separate FCR-resistance and salinity-response evaluations together with desirable agronomic characteristics, identifying candidate germplasm for further breeding and evaluation. The results also demonstrated that phenotypic responses to salinity differed between germination and early seedling growth, as germination performance did not consistently predict seedling responses under severe salinity stress. The generally weak associations among FCR resistance, salinity responses, and agronomic traits suggest that these traits represent largely independent dimensions of phenotypic variation, supporting their concurrent consideration in future breeding programs. However, because stress responses and agronomic performance were evaluated in separate experiments, and agronomic traits were assessed only under non-stress conditions, the identified genotypes should be regarded as candidate breeding germplasm rather than confirmed sources of field-level adaptation. Overall, this study provides a valuable phenotypic resource for triticale improvement and identifies candidate germplasm for further evaluation in breeding programs addressing multiple production constraints. Future field validation and molecular or genomic studies will be required to confirm the stability of these phenotypic responses and to investigate their underlying genetic basis.

##  Supplemental Information

10.7717/peerj.21736/supp-1 Supplemental Information 1 List of triticale genotypes and the wheat/rye controls investigated in this study

10.7717/peerj.21736/supp-2 Supplemental Information 2 One-way analysis of variance (ANOVA) for *Fusarium* crown rot disease index (DI) among triticale genotypes

10.7717/peerj.21736/supp-3 Supplemental Information 3 Analysis of germination percentage (GP) using a generalized linear model with binomial error distribution and logit link function Genotype, salt treatment, and their interaction were included as fixed effects. Significance was assessed using likelihood-ratio chi-square tests.

10.7717/peerj.21736/supp-4 Supplemental Information 4 Two-way analysis of variance (ANOVA) for relative germination rate (RGR) of triticale genotypes under 200 and 300 mM NaCl treatments Genotype, salt treatment, and their interaction were treated as fixed effects. RGR was calculated for each biological replicate using the corresponding control germination percentage. F-statistics and associated *P*-values are shown for each source of variation.

10.7717/peerj.21736/supp-5 Supplemental Information 5 Two-way analysis of variance (ANOVA) for seedling-stage salt injury index (SII) of triticale genotypes under 3 00 and 4 00 mM NaCl treatments Genotype, salt treatment, and their interaction were treated as fixed effects. SII was calculated for each biological replicate based on the seedling injury rating scale. F-statistics and associated *P*-values are presented for each source of variation. Analysis based on the 49 entries that reached the two-leaf stage and produced sufficient healthy seedlings for seedling-stage salinity evaluation.

10.7717/peerj.21736/supp-6 Supplemental Information 6 Agronomic trait values (mean ±SD) and multiple comparison results among triticale genotypes. Different letters indicate significant differences among genotypes at *P* < 0.05 based on Tukey’s HSD test

10.7717/peerj.21736/supp-7 Supplemental Information 7 Germination of triticale genotypes under control and salt stress (0, 200, and 300 mM NaCl) Each horizontal line represents a genotype; dots indicate mean ±standard deviation (n = 3) per treatment. Blue: 0 mM; orange: 200 mM; red: 300 mM. Genotypes are ranked by germination rate at 300 mM NaCl.

10.7717/peerj.21736/supp-8 Supplemental Information 8 Mean relative salt injury of triticale genotypes under salt stress (200 and 300 mM NaCl) at the germination stage Each horizontal line represents one genotype, with colored dots indicating mean RSI values at 200 mM (orange) and 300 mM (red) NaCl. Genotypes are ranked by RSI under 300 mM NaCl.

10.7717/peerj.21736/supp-9 Supplemental Information 9 Pearson correlation analysis among *Fusarium* crown rot resistance, salinity tolerance, and agronomic traits of triticale genotypes Pearson correlation coefficients were calculated among *Fusarium* crown rot disease index (FCR DI), germination percentage under control conditions (GR0), 200 mM NaCl (GR200), and 300 mM NaCl (GR300), relative germination rate at 200 mM (RGR200) and 300 mM NaCl (RGR300), seedling-stage salt injury index at 300 mM (SII300) and 400 mM NaCl (SII400), plant height (PH), spike length (SL), number of grains per spike (NGS), and thousand-kernel weight (TKW). Color intensity represents the magnitude and direction of Pearson correlation coefficients. Positive correlations are shown in blue and negative correlations in red. Significance levels are indicated as *P* < 0.05 (*), *P* < 0.01 (**), and *P* < 0.001 (***). Correlations were calculated using pairwise complete observations.

10.7717/peerj.21736/supp-10 Supplemental Information 10 Raw data (1) disease index scores from the Fusarium crown rot assays for all triticale genotypes; (2) germination rates under salt treatments; (3) relative salt injury at germination; (4) seedling-stage salt injury indices; and (5) agronomic trait measurements for all genotypes.

## Additional Information and Declarations

### Data Availability

The following information was supplied regarding data availability: The raw measurements are available in the Supplemental Files.
