# Supplementary material for: Phenotypic variation in Fusarium crown rot resistance and early-stage salt tolerance among triticale genotypes identifies candidate germplasm for breeding
Source: PeerJ. 2026 Sep 21;14:e21736. doi: 10.7717/peerj.21736 (PMC13603902; doi:10.7717/peerj.21736)

Relative salt injury of triticale genotypes under NaCl stress

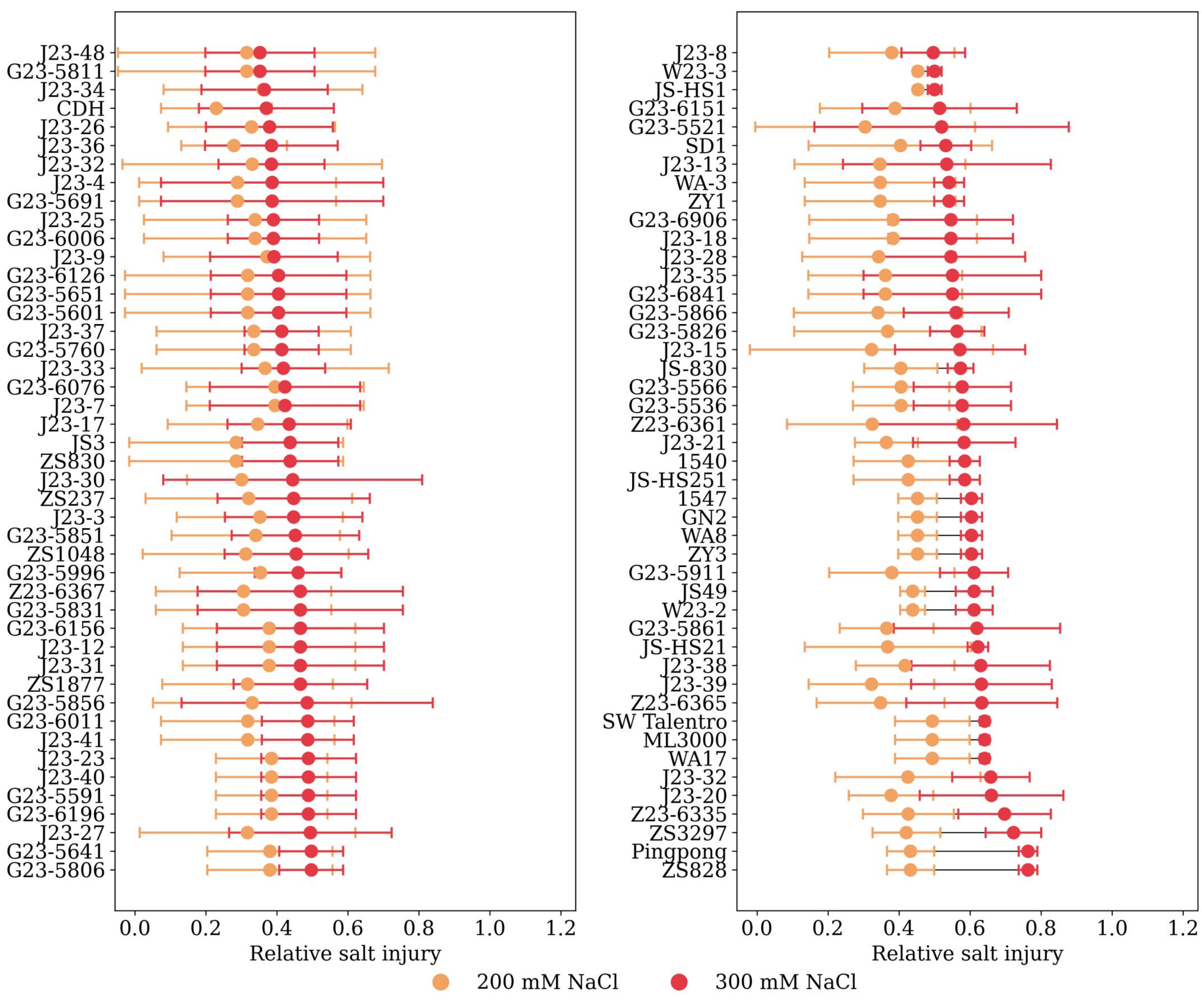

Supplement: Supplementary file 2 [file peerj-14-21736-s008.pdf]
