# Supplementary material for: Phenotypic variation in Fusarium crown rot resistance and early-stage salt tolerance among triticale genotypes identifies candidate germplasm for breeding
Source: PeerJ. 2026 Sep 21;14:e21736. doi: 10.7717/peerj.21736 (PMC13603902; doi:10.7717/peerj.21736)
Supplement: Supplementary file 3 [file peerj-14-21736-s009.pdf]

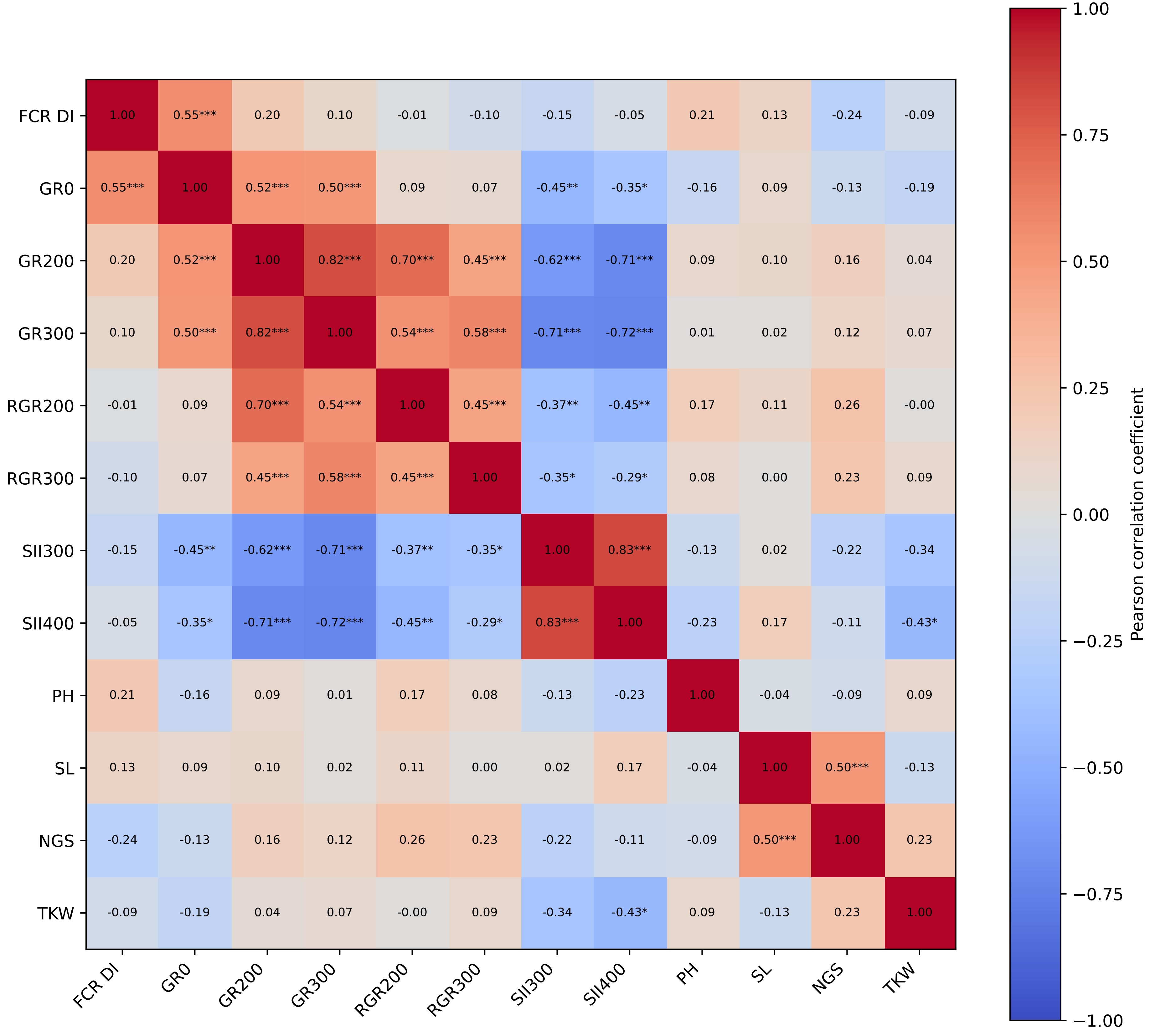
FCR DI 1.00 0.55*** 0.20 0.10 -0.01 -0.10 -0.15 -0.05 0.21 0.13 -0.24 -0.09
GR0 0.55*** 1.00 0.52*** 0.50*** 0.09 0.07 -0.45** -0.35* -0.16 0.09 -0.13 -0.19
GR200 0.20 0.52*** 1.00 0.82*** 0.70*** 0.45*** -0.62*** -0.71*** 0.09 0.10 0.16 0.04
GR300 0.10 0.50*** 0.82*** 1.00 0.54*** 0.58*** -0.71*** -0.72*** 0.01 0.02 0.12 0.07
RGR200 -0.01 0.09 0.70*** 0.54*** 1.00 0.45*** -0.37** -0.45** 0.17 0.11 0.26 -0.00
RGR300 -0.10 0.07 0.45*** 0.58*** 0.45*** 1.00 -0.35* -0.29* 0.08 0.00 0.23 0.09
SII300 -0.15 -0.45** -0.62*** -0.71*** -0.37** -0.35* 1.00 0.83*** -0.13 0.02 -0.22 -0.34
SII400 -0.05 -0.35* -0.71*** -0.72*** -0.45** -0.29* 0.83*** 1.00 -0.23 0.17 -0.11 -0.43*
PH 0.21 -0.16 0.09 0.01 0.17 0.08 -0.13 -0.23 1.00 -0.04 -0.09 0.09
SL 0.13 0.09 0.10 0.02 0.11 0.00 0.02 0.17 -0.04 1.00 0.50*** -0.13
NGS -0.24 -0.13 0.16 0.12 0.26 0.23 -0.22 -0.11 -0.09 0.50*** 1.00 0.23
TKW -0.09 -0.19 0.04 0.07 -0.00 0.09 -0.34 -0.43* 0.09 -0.13 0.23 1.00
FCR DI GR0 GR200 GR300 RGR200 RGR300 SII300 SII400 PH SL NGS TKW
1.00 0.75 0.50 0.25 0.00 −0.25 −0.50 −0.75 −1.00
Pearson correlation coefficient
