# Supplementary material for: Phenotypic variation in Fusarium crown rot resistance and early-stage salt tolerance among triticale genotypes identifies candidate germplasm for breeding
Source: PeerJ. 2026 Sep 21;14:e21736. doi: 10.7717/peerj.21736 (PMC13603902; doi:10.7717/peerj.21736)
Supplement: Supplementary file 4 [file peerj-14-21736-s001.docx]

**Table S1.** List of triticale genotypes and the wheat/rye controls investigated in this study

| **Genotype** | **Ploidy level** | **Description** |
| --- | --- | --- |
| CDH | Hexaploid | Salt tolerant wheat cultivar |
| W23-3 | Diploid | rye cultivar |
| WA-3 | Diploid | rye cultivar |
| WA8 | Diploid | rye cultivar |
| WA17 | Diploid | rye cultivar |
| JS49 | Octoploid | Triticale germplasm collection |
| ZY1 | Hexaploid | Triticale germplasm collection |
| ZY3 | Hexaploid | Triticale germplasm collection |
| JS-830 | Hexaploid | Triticale germplasm collection |
| JS-HS1 | Hexaploid | Triticale germplasm collection |
| JS-HS21 | Hexaploid | Triticale germplasm collection |
| JS-HS251 | Hexaploid | Triticale germplasm collection |
| 1540 | Hexaploid | Triticale germplasm collection |
| 1547 | Hexaploid | Triticale germplasm collection |
| Pingpong | Hexaploid | Triticale germplasm collection |
| SW Talentro | Hexaploid | Triticale germplasm collection |
| W23-2 | Hexaploid | Triticale germplasm collection |
| SD1 | Hexaploid | Triticale cultivar collection |
| ZS237 | Hexaploid | Triticale cultivar collection |
| ZS828 | Hexaploid | Triticale cultivar collection |
| ZS830 | Hexaploid | Triticale cultivar collection |
| ZS1048 | Hexaploid | Triticale cultivar collection |
| ZS1877 | Hexaploid | Triticale cultivar collection |
| ZS3297 | Hexaploid | Triticale cultivar collection |
| GN2 | Hexaploid | Triticale cultivar collection |
| JS3 | Hexaploid | Triticale cultivar collection |
| ML3000 | Hexaploid | Triticale cultivar collection |
| J23-32 | Hexaploid | Triticale advanced breeding line |
| J23-3 | Hexaploid | Triticale advanced breeding line |
| J23-4 | Hexaploid | Triticale advanced breeding line |
| J23-7 | Hexaploid | Triticale advanced breeding line |
| J23-8 | Hexaploid | Triticale advanced breeding line |
| J23-9 | Hexaploid | Triticale advanced breeding line |
| J23-12 | Hexaploid | Triticale advanced breeding line |
| J23-13 | Hexaploid | Triticale advanced breeding line |
| J23-15 | Hexaploid | Triticale advanced breeding line |
| J23-17 | Hexaploid | Triticale advanced breeding line |
| J23-18 | Hexaploid | Triticale advanced breeding line |
| J23-20 | Hexaploid | Triticale advanced breeding line |
| J23-21 | Hexaploid | Triticale advanced breeding line |
| J23-23 | Hexaploid | Triticale advanced breeding line |
| J23-25 | Hexaploid | Triticale advanced breeding line |
| J23-26 | Hexaploid | Triticale advanced breeding line |
| J23-27 | Hexaploid | Triticale advanced breeding line |
| J23-28 | Hexaploid | Triticale advanced breeding line |
| J23-30 | Hexaploid | Triticale advanced breeding line |
| J23-31 | Hexaploid | Triticale advanced breeding line |
| J23-32 | Hexaploid | Triticale advanced breeding line |
| J23-33 | Hexaploid | Triticale advanced breeding line |
| J23-34 | Hexaploid | Triticale advanced breeding line |
| J23-35 | Hexaploid | Triticale advanced breeding line |
| J23-36 | Hexaploid | Triticale advanced breeding line |
| J23-37 | Hexaploid | Triticale advanced breeding line |
| J23-38 | Hexaploid | Triticale advanced breeding line |
| J23-39 | Hexaploid | Triticale advanced breeding line |
| J23-40 | Hexaploid | Triticale advanced breeding line |
| J23-41 | Hexaploid | Triticale advanced breeding line |
| J23-48 | Hexaploid | Triticale advanced breeding line |
| G23-5521 | Hexaploid | Triticale advanced breeding line |
| G23-5536 | Hexaploid | Triticale advanced breeding line |
| G23-5566 | Hexaploid | Triticale advanced breeding line |
| G23-5591 | Hexaploid | Triticale advanced breeding line |
| G23-5601 | Hexaploid | Triticale advanced breeding line |
| G23-5641 | Hexaploid | Triticale advanced breeding line |
| G23-5651 | Hexaploid | Triticale advanced breeding line |
| G23-5691 | Hexaploid | Triticale advanced breeding line |
| G23-5760 | Hexaploid | Triticale advanced breeding line |
| G23-5806 | Hexaploid | Triticale advanced breeding line |
| G23-5811 | Hexaploid | Triticale advanced breeding line |
| G23-5826 | Hexaploid | Triticale advanced breeding line |
| G23-5831 | Hexaploid | Triticale advanced breeding line |
| G23-5851 | Hexaploid | Triticale advanced breeding line |
| G23-5856 | Hexaploid | Triticale advanced breeding line |
| G23-5861 | Hexaploid | Triticale advanced breeding line |
| G23-5866 | Hexaploid | Triticale advanced breeding line |
| G23-5911 | Hexaploid | Triticale advanced breeding line |
| G23-5996 | Hexaploid | Triticale advanced breeding line |
| G23-6006 | Hexaploid | Triticale advanced breeding line |
| G23-6011 | Hexaploid | Triticale advanced breeding line |
| G23-6076 | Hexaploid | Triticale advanced breeding line |
| G23-6126 | Hexaploid | Triticale advanced breeding line |
| G23-6151 | Hexaploid | Triticale advanced breeding line |
| G23-6156 | Hexaploid | Triticale advanced breeding line |
| G23-6196 | Hexaploid | Triticale advanced breeding line |
| Z23-6335 | Hexaploid | Triticale advanced breeding line |
| Z23-6361 | Hexaploid | Triticale advanced breeding line |
| Z23-6365 | Hexaploid | Triticale advanced breeding line |
| Z23-6367 | Hexaploid | Triticale advanced breeding line |
| G23-6841 | Hexaploid | Triticale advanced breeding line |
| G23-6906 | Hexaploid | Triticale advanced breeding line |
