# Supplementary material for: Phenotypic variation in Fusarium crown rot resistance and early-stage salt tolerance among triticale genotypes identifies candidate germplasm for breeding
Source: PeerJ. 2026 Sep 21;14:e21736. doi: 10.7717/peerj.21736 (PMC13603902; doi:10.7717/peerj.21736)
Supplement: Supplementary file 5 [file peerj-14-21736-s002.docx]

**Table S2.** One-way analysis of variance (ANOVA) for *Fusarium* crown rot disease index (DI) among triticale genotypes.

| **Source of variation** | **df** | **Mean square** | **F value** | ***P* value** |
| --- | --- | --- | --- | --- |
| Genotype | 84 | 1306.78 | 6.57 | <0.001 |
| Error | 130 | 198.94 |  |  |
