# Supplementary material for: Phenotypic variation in Fusarium crown rot resistance and early-stage salt tolerance among triticale genotypes identifies candidate germplasm for breeding
Source: PeerJ. 2026 Sep 21;14:e21736. doi: 10.7717/peerj.21736 (PMC13603902; doi:10.7717/peerj.21736)
Supplement: Supplementary file 6 [file peerj-14-21736-s003.docx]

**Table S3.** Analysis of germination percentage (GP) using a generalized linear model with binomial error distribution and logit link function.

| **Source of variation** | **df** | **χ²** | ***P* value** |
| --- | --- | --- | --- |
| Genotype | 89 | 645.56 | <0.001 |
| Salt treatment | 2 | 2979.47 | <0.001 |
| Genotype × Salt | 178 | 391.5 | <0.001 |

Genotype, salt treatment, and their interaction were included as fixed effects. Significance was assessed using likelihood-ratio chi-square tests.
