# Supplementary material for: Phenotypic variation in Fusarium crown rot resistance and early-stage salt tolerance among triticale genotypes identifies candidate germplasm for breeding
Source: PeerJ. 2026 Sep 21;14:e21736. doi: 10.7717/peerj.21736 (PMC13603902; doi:10.7717/peerj.21736)
Supplement: Supplementary file 7 [file peerj-14-21736-s004.docx]

**Table S4.** Two-way analysis of variance (ANOVA) for relative germination rate (RGR) of triticale genotypes under 200 and 300 mM NaCl treatments.

| **Source of variation** | **df** | **Mean square** | **F value** | ***P* value** |
| --- | --- | --- | --- | --- |
| Genotype | 89 | 3993.74 | 38.85 | <0.001 |
| Salt treatment | 1 | 2293.79 | 22.31 | <0.001 |
| Genotype × Salt | 89 | 179.56 | 1.75 | <0.001 |

Genotype, salt treatment, and their interaction were treated as fixed effects. RGR was calculated for each biological replicate using the corresponding control germination percentage. F-statistics and associated *P*-values are shown for each source of variation.
