# Supplementary material for: Phenotypic variation in Fusarium crown rot resistance and early-stage salt tolerance among triticale genotypes identifies candidate germplasm for breeding
Source: PeerJ. 2026 Sep 21;14:e21736. doi: 10.7717/peerj.21736 (PMC13603902; doi:10.7717/peerj.21736)
Supplement: Supplementary file 8 [file peerj-14-21736-s005.docx]

**Table S5.** Two-way analysis of variance (ANOVA) for seedling-stage salt injury index (SII) of triticale genotypes under 300 and 400 mM NaCl treatments.

| **Source of variation** | **df** | **Mean square** | **F value** | ***P* value** |
| --- | --- | --- | --- | --- |
| Genotype | 48 | 917.79 | 156.74 | <0.001 |
| Salt treatment | 1 | 22651.97 | 3868.59 | <0.001 |
| Genotype × Salt | 48 | 111.55 | 19.05 | <0.001 |

Genotype, salt treatment, and their interaction were treated as fixed effects. SII was calculated for each biological replicate based on the seedling injury rating scale. F-statistics and associated *P*-values are presented for each source of variation. Analysis based on the 49 entries that reached the two-leaf stage and produced sufficient healthy seedlings for seedling-stage salinity evaluation.
