# Supplementary material for: Phenotypic variation in Fusarium crown rot resistance and early-stage salt tolerance among triticale genotypes identifies candidate germplasm for breeding
Source: PeerJ. 2026 Sep 21;14:e21736. doi: 10.7717/peerj.21736 (PMC13603902; doi:10.7717/peerj.21736)
Supplement: Supplementary file 9 [file peerj-14-21736-s006.docx]

**Table S6.** Agronomic trait values (mean ± SD) and multiple comparison results among triticale genotypes. Different letters indicate significant differences among genotypes at *P* < 0.05 based on Tukey’s HSD test.

| Genotype | Plant Height (cm) | Group | Spike length (cm) | Group | Number of grains per spike | Group | Thousand kernel weight (g) | Group |
| --- | --- | --- | --- | --- | --- | --- | --- | --- |
| ZS1048 | 163.8 ± 1.16 | defghi | 15.8 ± 1.32 | a | 32 ± 1.73 | ab | 31.6 ± 1.53 | f |
| ZS830 | 157.4 ± 1.73 | ijklmno | 10 ± 0.76 | fg | 22.6 ± 2.00 | mn | 34.32 ± 1.53 | e |
| ZS1877 | 162.4 ± 2.08 | efghij | 12.3 ± 0.29 | bcdef | 24 ± 1.53 | klmn | 36 ± 1.15 | e |
| JS3 | 164.8 ± 1.53 | cdefgh | 12.6 ± 0.58 | bcdef | 32.4 ± 0.58 | a | 40.68 ± 1.00 | de |
| ZS237 | 177.6 ± 1.16 | a | 11.9 ± 0.58 | bcdef | 27.6 ± 1.00 | defghijklm | 37.92 ± 1.15 | e |
| J23-3 | 161.4 ± 1.53 | fghijkl | 11.4 ± 1.04 | bcdef | 25.2 ± 1.53 | ghijklmn | 35.24 ± 1.15 | e |
| J23-4 | 158.8 ± 2.52 | hijklmn | 12.2 ± 0.50 | bcde | 29.2 ± 1.53 | abcdefghi | 39.24 ± 0.58 | de |
| J23-7 | 164.6 ± 1.16 | bcdefg | 11.1 ± 0.58 | cdefg | 26.4 ± 1.73 | fghijklmn | 36.48 ± 1.15 | e |
| J23-8 | 158.8 ± 2.08 | ijklmno | 12.9 ± 1.00 | abc | 31 ± 0.58 | abcde | 37.8 ± 1.15 | e |
| J23-9 | 164.2 ± 1.16 | defghi | 12.1 ± 0.50 | bcde | 23.6 ± 1.73 | mn | 36.72 ± 1.53 | e |
| J23-12 | 161.2 ± 1.53 | fghijkl | 12.3 ± 0.76 | bcdef | 23.4 ± 0.58 | lmn | 36.28 ± 1.53 | e |
| J23-13 | 154.2 ± 2.52 | lmnop | 10.8 ± 0.76 | defg | 26 ± 0.58 | ghijklmn | 36.2 ± 1.53 | e |
| J23-15 | 163.4 ± 0.58 | efghij | 13.1 ± 1.04 | bcd | 29.8 ± 0.58 | abcde | 38.36 ± 1.53 | e |
| J23-17 | 134.8 ± 1.53 | r | 13.1 ± 0.87 | abc | 30.6 ± 1.53 | abc | 37.92 ± 1.15 | e |
| J23-18 | 132 ± 2.52 | r | 12 ± 1.26 | bcdef | 29 ± 1.73 | bcdefghij | 37.8 ± 1.53 | e |
| J23-20 | 151.2 ± 1.53 | opq | 13.3 ± 1.26 | abc | 24.4 ± 1.13 | klmn | 35.48 ± 1.53 | e |
| J23-21 | 152.6 ± 0.58 | nopq | 11.7 ± 0.50 | bcdef | 22.4 ± 0 | mn | 43.48 ± 2.87 | a |
| J23-23 | 154 ± 1.53 | mnop | 10.1 ± 0.76 | fg | 23.2 ± 0.58 | lmn | 35.76 ± 1.67 | e |
| J23-25 | 154.6 ± 2.00 | lmnop | 12.6 ± 0.76 | bcde | 30.8 ± 1.00 | abcd | 41.32 ± 2.06 | bcde |
| J23-26 | 156 ± 0.58 | klmno | 11.8 ± 0.76 | bcdef | 28.8 ± 1.00 | abcdefgh | 37.22 ± 1.21 | e |
| J23-27 | 162.2 ± 1.53 | fghijkl | 10.9 ± 0.76 | cdefg | 24.4 ± 1.16 | ijklmn | 41.6 ± 1.76 | abc |
| J23-28 | 162.4 ± 1.00 | fghijk | 12.3 ± 0.76 | bcdef | 28 ± 1.16 | cdefghijk | 37.92 ± 1.67 | e |
| J23-30 | 158.8 ± 1.53 | hijklmn | 12.7 ± 0.50 | abc | 29 ± 0.58 | abcdefg | 40.56 ± 2.06 | bcde |
| J23-31 | 152.6 ± 1.53 | opq | 11.3 ± 0.76 | cdefg | 26.4 ± 1.00 | defghijklm | 35.94 ± 1.21 | e |
| J23-32 | 157.8 ± 1.53 | hijklmn | 12.1 ± 0.58 | bcdef | 26.8 ± 1.53 | efghijklm | 42.48 ± 1.76 | abc |
| J23-33 | 163.6 ± 1.53 | defghi | 10.1 ± 0.76 | cdefg | 28.2 ± 1.53 | bcdefghi | 39.06 ± 1.49 | cde |
| J23-34 | 148.8 ± 0.58 | pq | 11.8 ± 0.76 | bcdef | 28.8 ± 0.58 | abcdefgh | 43.8 ± 3.45 | ab |
| J23-35 | 157.8 ± 1.53 | jklmno | 11.9 ± 0.50 | cdef | 25.4 ± 0.58 | fghijklmn | 42.94 ± 2.71 | a |
| J23-36 | 153.2 ± 2.00 | nopq | 10.9 ± 0.29 | cdefg | 27.2 ± 1.53 | defghijkl | 45.66 ± 3.06 | a |
| J23-37 | 165.4 ± 3.00 | bcdefg | 11.3 ± 0.76 | defg | 28.6 ± 1.16 | abcdefgh | 41.88 ± 1.21 | abcd |
| J23-38 | 146.2 ± 2.52 | q | 11 ± 0.76 | cdefg | 29.4 ± 1.00 | abcdefgh | 43.8 ± 1.76 | abc |
| J23-39 | 171.6 ± 1.00 | ab | 10.7 ± 0.58 | cdefg | 27.2 ± 1.16 | ghijklmn | 39.46 ± 1.49 | cde |
| J23-40 | 167.4 ± 0.58 | bcdef | 9.1 ± 0.58 | g | 22 ± 0.58 | n | 41.04 ± 3.45 | ab |
| J23-41 | 170.8 ± 1.53 | bcd | 11.7 ± 0.76 | bcdef | 29 ± 1.53 | abcdefg | 44.26 ± 2.71 | a |
| J23-48 | 165 ± 1.16 | defghi | 13.7 ± 0.29 | ab | 31.4 ± 1.16 | abc | 46.9 ± 3.06 | a |
| G23-5521 | 165 ± 3.51 | bcdef | 11.6 ± 0.50 | cdef | 23.4 ± 1.73 | mn | 40.24 ± 1.21 | abcd |
| G23-5536 | 169.2 ± 3.61 | bcde | 12.2 ± 0.50 | bcde | 24.2 ± 1.00 | jklmn | 42.5 ± 2.71 | a |
| G23-5566 | 169.4 ± 3.51 | bcde | 11.4 ± 0.58 | bcdef | 23.2 ± 1.73 | mn | 44.06 ± 3.06 | a |
| G23-5591 | 171 ± 2.52 | ab | 10.9 ± 0.76 | cdefg | 23.4 ± 0.58 | lmn | 40.44 ± 1.21 | abcd |
| G23-5601 | 157 ± 3.51 | jklmno | 11.8 ± 0.50 | bcde | 25 ± 0.58 | ghijklmn | 41.72 ± 1.76 | abc |
| G23-5641 | 155.6 ± 2.08 | mnop | 10.9 ± 0.76 | cdefg | 22 ± 0.58 | n | 36.62 ± 1.49 | cde |
| G23-5651 | 159.6 ± 2.08 | ghijklm | 13.4 ± 0.50 | abc | 25.6 ± 1.16 | hijklmn | 42.96 ± 3.45 | ab |
| G23-5691 | 167.4 ± 2.52 | bcdef | 11.2 ± 0.29 | bcdef | 29.8 ± 1.00 | abcdef | 46.36 ± 2.87 | a |
| G23-5761 | 170.8 ± 2.52 | bc | 12.8 ± 1.76 | bcd | 28.2 ± 1.73 | bcdefghij | 38.36 ± 1.67 | e |
| G23-5806 | 170.8 ± 1.53 | bc | 11.3 ± 0.29 | cdefg | 23.6 ± 1.16 | klmn | 38.68 ± 2.06 | bcde |
| G23-5811 | 169.4 ± 3.06 | bcde | 10.9 ± 0.76 | efg | 22 ± 1.16 | n | 34.46 ± 1.21 | e |
| Z23-6361 | 158.8 ± 2.00 | ijklmno | 11.7 ± 0.76 | cdefg | 27.8 ± 1.00 | abcdefgh | 42.48 ± 1.76 | abc |
| Z23-6365 | 152.8 ± 3.06 | mnopq | 12.9 ± 1.00 | abc | 24.8 ± 0.58 | hijklmn | 28.56 ± 1.53 | f |
